# The therapeutic alliance during remotely delivered therapy: A Delphi study with health professionals

**DOI:** 10.1111/bjc.12544

**Published:** 2025-04-04

**Authors:** Sam Tyrrell, Sandra Bucci, Katherine Berry

**Affiliations:** ^1^ Division of Psychology and Mental Health, School of Health Sciences, Faculty of Biology, Medicine and Health, Manchester Academic Health Sciences Centre The University of Manchester Manchester UK; ^2^ Department of Research and Innovation Greater Manchester Mental Health NHS Foundation Trust Manchester UK

**Keywords:** alliance, Delphi, online therapy, remote therapy, telephone, videoconferencing

## Abstract

**Objectives:**

Delivering psychological therapy via videoconferencing and telephone is now commonplace across mental health services, but many therapists remain concerned about the impact on the therapeutic alliance. This study aimed to establish consensus amongst psychological therapists regarding the factors involved in establishing and maintaining the therapeutic alliance during remote therapy interventions.

**Methods:**

Psychological therapists from a range of professional backgrounds were invited to complete a three‐Round Delphi survey online. Round 1 generated qualitative data which was used to develop a list of statements relating to key factors in establishing and maintaining alliance in therapy delivered over the telephone or videoconferencing. Participants were invited to rate their level of agreement with these statements in Rounds 2 and 3.

**Results:**

Of the 149 participants who completed Round 1, 93 completed Round 2, and 71 participants completed all three Rounds. Following Round 3, a high level of agreement (above 80%) was obtained in relation to 31/63 statements reflecting communication style, contracting, quality and value, environment, emotional differences, effort, and technological aspects of engaging clients in this way. Participants reported similar views for therapies delivered via telephone and videoconferencing.

**Discussion:**

Clinicians who have had to navigate the rapid rise in online delivery of therapy have valuable insights which warrant sharing amongst communities of practicing therapists and those in training. Identifying factors which therapists agree are important in developing alliances with patients remotely also guides researchers in identifying factors that warrant further investigation through empirical studies.

## INTRODUCTION

The benefits of remotely delivered psychological therapies in terms of cost savings, convenience, and improving accessibility were recognized even prior to the COVID‐19 pandemic (Connolly et al., 2020). The pandemic pushed forward the digital agenda across health services worldwide, and psychological therapy delivered either via telephone or video conferencing is now commonplace in mental health services and is heralded as a means of maximizing service user choice (Hanley, 2020; Kotera et al., 2021). Nonetheless, some therapists report reservations about remotely delivered therapy due to concerns about the impact of the remote medium on the development of a therapeutic alliance (Kotera et al., 2021; James et al., 2022; Full et al., 2024) and rate the therapeutic alliance lower when delivering remote compared with in‐person therapy (Eichenberg et al., 2022). Therapists' concerns about alliance within remotely delivered therapies may relate to their knowledge that therapeutic alliance predicts therapy outcomes (Baier et al., 2020; Martin et al., 2000). Despite therapist lower ratings of alliance in therapy and their consequent concerns about outcomes, research suggests that the client's perception of the quality of therapeutic relationships remains comparable or even superior in remotely delivered therapy compared with therapy delivered in‐person (Eichenberg et al., 2022; Jenkins‐Guarnieri et al., 2015). The evidence also suggests that remotely delivered therapy is effective as therapy delivered in‐person (Brenes et al., 2011; Greenwood et al., 2022; Thomas et al., 2021). For example, Greenwood et al. (2022) conducted a systematic review and meta‐analysis of studies comparing therapy outcomes for common mental health conditions and physical health conditions in face‐to‐face therapy with therapies delivered via video or telephone. The authors reviewed 12 randomized controlled trials and found no significant differences in outcomes across the different modes of delivery. Therapeutic alliance was comparable in five of the studies that measured alliance (three from the client perspective and two from the therapist perspective), although heterogeneity statistics were high, suggesting findings across the studies differed.

The evidence withstanding, it is important to ensure that therapists feel competent in delivering therapy through a range of different mediums that are used within mental health services. Whilst guidelines exist for the delivery of therapy remotely (American Psychological Associate (APA), 2013; British Psychological Society (BPS), 2020), these do not make explicit reference to the formation of therapeutic alliance. There is a large research literature on factors that promote the development and maintenance of therapeutic alliance in therapy, which includes therapist factors, such as level of empathy and warmth (Ryan et al., 2023) and a wide range of client‐related factors, ranging from interpersonal variables such as attachment styles (Smith et al., 2010) to psychiatric diagnoses (Barr et al., 2023). However, the rapid growth of remotely delivered therapies over recent years has meant that the research data are lagging behind clinical practice. Therapists have therefore had to navigate the alliance formation within remotely delivered therapies through their own clinical practice. Practice‐based learning is invaluable in skill development (Beidas & Kendall, 2010) and knowledge generated through these experiences is therefore important to capture and share. This Delphi study identified the opinions and views of psychological therapists involved in delivering remotely psychological therapy on the process of establishing and maintaining a therapeutic alliance during remotely delivered psychological therapies. This study also aimed to explore any differences in establishing and maintaining a therapeutic alliance when therapy is delivered remotely compared with therapy in person.

## METHODS

The study was approved by the University of Manchester Research Ethics Committee (ref: 2021‐12957‐21208) and was conducted during the year of 2022.

### Design

The Delphi method involves multiple ‘Rounds’ where anonymous experts or panellists are asked to provide their opinions to obtain consensus on a topic (Keeney et al., 2006). Panellists are asked to rate their agreement with statements and are encouraged during this process to move toward consensus with their fellow participants. This Delphi study involved three Rounds. In Round 1, participants were invited to answer a set of open‐ended survey questions about developing a therapeutic alliance within remotely delivered therapies. These responses were grouped and used to generate statements for rating in terms of level of agreement in Round 2. In Round 3, participants were asked to adjust their initial ratings of statements in relation to the responses of other participants where agreement was not reached.

### Participants

Inclusion criteria were: (i) an academic qualification at least at undergraduate level recognized by the BPS, British Association for Counselling and Psychotherapy or the British Association for Counselling (to ensure participants had a core level of psychological knowledge whilst allowing professionals from diverse professional backgrounds to take part); (ii) responding affirmatively when asked to confirm that the following statement accurately reflected their professional practice: *psychological therapy is defined as a patient meeting with a therapist (a healthcare professional competent in giving psychological therapy) to talk about their feelings and thoughts and how these affect behaviour and well‐being* (Adapted from, National Institute for Care Excellence, 2014) (to ensure that participants were actively engaged in the delivery of psychological therapy); (iii) had delivered remote synchronous therapy sessions (i.e. delivery of therapy to patients in real‐time through a telephone or an online virtual platform that support video and audio communication, for example, Microsoft Teams, FaceTime) over the past 24 months. The timeframe of 24 months was used to ensure that participants were able to reflect on the changes in practice during the COVID‐19 pandemic and beyond.

Participants were recruited either via advertisements posted on online social media platforms [e.g. Facebook; X (formerly Twitter)] with no restrictions placed on geographical location, or via email sent to practitioners working in private sector clinics or enrolled in a mental health‐related training course in the United Kingdom. No restrictions were placed on the patient population that practitioners worked with or the therapy models practitioners used.

There is no agreement on the required sample size for Delphi studies, although studies typically range from 10 to 100 participants (Akins et al., 2005). Given that multiple Round online Delphi studies often have high rates of attrition (Hall et al., 2018), we aimed to recruit at least 100 participants at Round 1.

### Procedure

Participants were provided with a web link to access the Delphi survey, which included information about the study, a brief questionnaire asking about job title and the approximate number of patients they had delivered remote psychological therapy to, and a consent form. The survey was hosted on the Qualtrics platform. Following consent, participants were assigned an identification number, which was linked to their email address (used to contact participants in subsequent Rounds) in a separate document. Participants were given up to 4 weeks to complete each Round. After the first and second Rounds, an email was sent out with a web link to access and complete the subsequent Round. Participants were entered into a draw to win one of eight £50 Amazon vouchers following the completion of all three Delphi rounds. To assess consensus of responses to items generated following Round 1, the predefined percentage of agreement was set at 80% (Langlands et al., 2008).

### Round 1 questionnaire

Round 1 comprised seven open‐ended questions designed to ascertain views on engaging patients in remote synchronous therapy with a specific focus on establishing and maintaining the therapeutic alliance. Questions were generated through discussion within the research team and broadly asked participants to reflect on times when alliance building went well and times when it went poorly, the differences between videoconferencing and telephone mediums, and the differences between remote and face‐to‐face interventions. Qualitative content analysis was used identify relevant themes in responses and was achieved by categorizing words, phrases and paragraphs into units of analysis that convey a similar central meaning or theme (Graneheim & Lundman, 2004; Robson, 2011). Data were exported from Qualtrics to a Microsoft Excel spreadsheet to support analysis. Data were categorized into preliminary codes which were then grouped into broader concepts. Similarities between codes and concepts were identified and codes were combined into sub‐categories. Each question was addressed in order; that is, question one responses for all participants were coded before moving onto question two. In this way, question‐specific codes were established, and the bank of codes grew as data from each question was analysed. These subthemes were then reviewed alongside the data by all three members of the research team and overarching themes were derived. Discussion within the research team ensured that the themes were defined and named in a way that captured the essence of the data it represented. Following a process outlined by Strauss and Corbin (1998), a range of statements were then listed in relation to each theme with care taken to ensure that the statements reflected different aspects of the theme but were also ‘actionable’ (data that referred to specific actions that could be taken by participants that could influence the therapeutic relationship).

### Round 2 questionnaire

The results of the Round 1 content analysis were used to produce the items for the Round 2 survey which asked about factors important for the development of therapeutic alliance during remotely delivered synchronous therapies. Before completing this survey Round, participants were presented with filter questions that asked about whether they delivered therapy over the telephone or via video conferencing or both. Based on their responses to this filter question, a set of questions specific to telephone or video modes of delivery or both sets of questions were displayed. Participants rated the strength of agreement against each statement on a 5‐point Likert scale (1 = strongly agree; 2 = somewhat agree; 3 = neither agree nor disagree; 4 = somewhat disagree; 5 = strongly disagree). Participants were also given the option to respond, ‘Don't know’. Both responses ‘strongly agree’ and ‘somewhat agree’ and ‘strongly disagree’ and ‘somewhat disagree’ were combined when calculating levels of agreement. The ‘neither agree nor disagree’ response was considered as a stand‐alone category. For example, if at least 80% of participants selected either strongly agree or somewhat agree in relation to a statement then it would be deemed to reach adequate consensus and labelled a ‘consensus statement’. Statements that did not achieve this level of agreement were carried forward into Round 3 for further rating.

### Round 3 questionnaire

The Round 3 survey included a summary of the findings from Round 2, including a list of established consensus statements. The statements achieving consensus were then presented alongside those statements that had not reached consensus, along with the participant's original response to each statement from Round 2 and a summary of participants' responses from Round 2 for each statement, presented as percentages. Participants were asked to re‐rate each statement and were informed that they could change their response based on the new information available to them or select the same response as they had in the previous Round. The same 80% criterion for consensus from Round 2 was also applied in Round 3. Additional statements that met the consensus threshold in Round 3 were added to the list of consensus statements from Round 2. Statements that did not reach consensus were excluded.

## RESULTS

### Sample summary

In total, 346 people clicked the link to the online survey, but only 149 participants completed Round 1. Participants reported a range of job titles reflecting the broad range of professionals who deliver psychological therapy, but the majority were clinical psychologists (*n* = 77) or trainee clinical psychologists (*n* = 43). Participants also varied in how many patients they had delivered remote psychological therapy to, from 1–10 patients (*n* = 47 participants) to >50 patients (*n* = 36 participants). Of the 149 participants who completed Round 1, 93 participants completed the Round 2 survey (38% attrition). This high level of attrition is common in Delphi methodology, with the literature reporting varied levels of participant retention, often between 20% and 87% (Hall et al., 2018). A further attrition rate of 24% in Round 3 resulted in 71 participants completing all three rounds.

### Round 1

Content analysis of participants' responses to Round 1 questions generated 30 sub‐categories that were grouped into seven overarching categories: (1) communication style (e.g. appropriate pacing is also key when online as it is harder to read body language cues, so making sure I give space/time for them to think and not try and fill the silence gaps); (2) contracting (e.g. naming potential difficulties and discussing options in internet connection drops or there is not a safe, private space etc.); (3) emotional differences (e.g. I had a client who continued to feel a bit anxious about using the online platform as they were not confident in using the technology. I think they would have felt less anxious face‐to‐face); (4) quality/value (e.g. I think the online forum meant it was more easily forgotten about and the quality of the sessions were affected as well); (5) environment (e.g. the client was older and was living in an unhappy marital relationship. Engaging with therapy whilst being in the home where there was so much unhappiness did not feel like the safe escape they had previously experienced within therapy); (6) effort (e.g. I need to concentrate intensely in order to pick up nuances and ensure that I understand the client/s); and (7) technology (e.g. It can also be more difficult to present materials sometimes and send information needed for the sessions or between session tasks, which you wouldn't have to deal with when face‐to‐face). Thirty‐three statements related to remote synchronous therapy via videoconferencing and 30 statements related to remote synchronous therapy via telephone were produced from open‐ended responses and presented to participants in Round 2 (see Appendix S1).

### Rounds 2 and 3

Twenty‐two out of 63 statements reached consensus (>80%) in Round 2; nine statements related to videoconferencing and 13 statements related to telephone as a means of delivering therapy. A further nine statements reached consensus following re‐rating in Round 3; six statements related to videoconferencing and three statements related to telephone as a means of delivering therapy. Following the completion of both Rounds, consensus was reached for 31 out of a total of 63 statements (15 statements about videoconferencing and 16 statements about telephone delivered therapy). Consensus levels ranged between 80% and 100% across items. See Table 1 for consensus statements and the round in which they reached consensus.

**TABLE 1:** Categories and items that reached >80% consensus in Round 2 and 3.

Items	Mean	SD	Variance	Consensus level %	Round consensus reached
Videoconferencing‐related statements					
Communication style					
It is important to use more listening sounds like ‘hmm’ and ‘ahh’ when working via online videoconferencing compared with face‐to‐face therapy	2.19	.96	.92	81.43	Round 3
Contracting					
It is important to allow the client to make the choice to access therapy via online videoconferencing as an alternative to face‐to‐face therapy	1.39	.68	.46	93.33	Round 2
It is important to continue to name difficulties with therapy via online videoconferencing as they occur	1.41	.67	.45	95.46	Round 2
It is important to explicitly acknowledge unintentionally interrupting clients during therapy via online videoconferencing	1.53	.86	.74	87.77	Round 2
It is important to develop a plan for when technology goes wrong during therapy when working via online videoconferencing	1.09	.28	.08	100.00	Round 2
It is important to take time to discuss the limitations of therapy via online videoconferencing with the client at the start of online therapy	1.64	.85	.72	91.43	Round 3
Emotional differences					
It is important to discuss the potential impact of seeing one's own face on screen during therapy via online videoconferencing	2.11	.67	.44	82.86	Round 3
Quality/Value					
It is important to discuss with the client whether they feel that therapy via online videoconferencing is as valuable as face‐to‐face therapy	1.81	.87	.76	82.03	Round 2
Environment					
It is important to discuss the appropriateness of the environment in which the client accesses therapy via online videoconferencing	1.14	.44	.19	98.89	Round 2
It is more important to discuss the confidentiality of the therapy environment when working via online videoconferencing compared with face‐to‐face	1.44	.88	.78	88.89	Round 2
It is important to discuss whether the client feels safe in the environment in which they are accessing therapy via online videoconferencing	1.18	.55	.30	97.77	Round 2
It is important to ensure that your (the professional) environment is consistent (i.e. the same location with the same background) when engaging in therapy via online videoconferencing	2.24	.85	.73	81.43	Round 3
It is important to notice and name distractions in the client's environment when working via online videoconferencing compared with face‐to‐face therapy	1.96	.57	.33	91.42	Round 3
Technology					
It is important to ensure that screen share is used to supplement communication during therapy via online videoconferencing, such as when drawing out formulations or sharing resources	1.66	.89	.79	86.51	Round 2
It is important to arrange additional time prior to the start of therapy via online videoconferencing to ensure that clients have the knowledge to use the technology to access therapy via videoconferencing	1.76	.93	.87	87.14	Round 3
Telephone‐related statements					
Communication style					
It is important to use more listening sounds like ‘hmm’ and ‘ahh’ when working via telephone.	1.27	.64	.41	96.00	Round 2
It is important to discuss the function of therapeutic silence explicitly during therapy when working via telephone	1.92	1.03	1.06	80.00	Round 2
It is important to seek more regular feedback about the client's experience of therapy when working via telephone compared with face‐to‐face therapy	1.71	.91	.82	85.34	Round 2
Contracting					
It is important to allow the client to make the choice to access therapy via telephone as an alternative to face‐to‐face therapy	1.60	.97	.93	86.67	Round 2
It is important to take time to discuss the limitations of therapy via telephone with the client at the start of telephone therapy	1.57	.73	.54	93.33	Round 2
It is important to continue to name difficulties with therapy via telephone as they occur	1.39	.59	.34	97.33	Round 2
It is important to explicitly acknowledge unintentionally interrupting clients during therapy via telephone	1.33	.62	.38	94.66	Round 2
It is important to develop a plan for when technology goes wrong during therapy when working via telephone	1.16	.40	.16	98.66	Round 2
Emotional differences					
It is important to discuss with the client the anonymity that comes when working via telephone which is often not present when working face‐to‐face or via videoconferencing software	1.91	.70	.49	86.20	Round 3
Quality/value					
It is important to discuss with the client whether they feel that therapy via telephone is as valuable as face‐to‐face therapy	1.62	.82	.67	86.49	Round 2
Environment					
It is important to discuss the appropriateness of the environment in which the client accesses therapy via telephone	1.19	.45	.21	97.33	Round 2
It is more important to discuss the confidentiality of the therapy environment when working via telephone compared with face‐to‐face	1.29	.65	.42	96.00	Round 2
It is important to discuss whether the client feels safe in the environment in which they are accessing therapy via telephone	1.15	.53	.29	98.66	Round 2
It is more important to notice and name distractions in the client's environment when working via telephone compared with face‐to‐ face therapy	1.93	.96	.92	81.33	Round 2
Effort					
It is important to acknowledge with the client that therapy via telephone is often more effortful than when working face‐to‐face	2.16	.74	.54	82.75	Round 3
Technology					
It is more important to send additional materials for use between therapy sessions via email (or appropriate alternative) when working via telephone compared with providing materials for use between sessions during face‐to‐face therapy	1.83	.99	.97	82.76	Round 3

#### Communication style

Participants reached consensus on three statements relating to the medium of telephone and one statement relating to videoconferencing. Participants agreed that listening sounds such as ‘hmm’ and ‘ahh’ positively impacted the therapeutic alliance through both remote mediums. Further statements relating to the telephone medium indicated the importance of discussing the function of therapeutic silence and seeking more regular feedback from patients about their experience of therapy compared with when working face‐to‐face.

#### Contracting

Participants reached consensus on the same five items for both therapy via telephone and videoconferencing. Participants agreed that during the contracting stage of therapy, it was important for the patient to make the choice to access therapy through remote means as an alternative to meeting face‐to‐face delivery, that it was important to develop a plan for when technology fails or goes wrong, and that it was important to name difficulties caused by the remote communication setting as they occur. Participants also felt that discussions whilst contracting should include the possibility that clinicians and patients may unintentionally interrupt each other and limitations related to engaging in therapy via both telephone and videoconferencing.

#### Emotional differences

Participants reached consensus on one statement under both the videoconferencing and telephone media. These were examples of medium‐specific statements (statements only relevant to one medium, rather than both). Participants indicated, in the case of videoconferencing, that it is important to discuss the potential impact of seeing one's own face on screen and, in the case of telephone therapy, participants agreed that it is important to discuss the anonymity afforded by telephone contact.

#### Quality/Value

Participants agreed that for both videoconferencing and telephone media, a discussion about the perceived value of therapy via remote means compared with face‐to‐face therapy would be important to the therapeutic alliance.

#### Environment

Statements concerning the therapy environment showed similar results across both mediums; five statements reached consensus for the videoconferencing medium and four for the telephone medium. Four of the statements were identical for both videoconferencing and telephone. These indicated the importance of ensuring that the patient's environment is appropriate, confidential, safe, and highlighted the importance of noticing and naming distractions in the patient's environment as they occur. Participants also indicated that for the videoconferencing medium alone, it is important to ensure that the professional's environment remains consistent in terms of location and background when engaging in therapy.

#### Effort

One statement reached consensus in relation to the telephone medium of delivering therapy, suggesting that it is important to acknowledge with the patient that therapy via telephone is often more effortful than face‐to‐face therapy.

#### Technology

Two statements reached consensus for videoconferencing. Participants indicated that it is important to use screen sharing software to supplement communication and that it is important to arrange additional time prior to the start of therapy to familiarize the patient with using videoconferencing technology. Regarding the telephone medium, participants highlighted the added importance of sending materials for use between sessions either by email or another appropriate medium compared with face‐to‐face therapy.

## DISCUSSION

The primary aim of this study was to seek consensus on clinicians' views about the factors involved in establishing and maintaining the therapeutic alliance when delivering remote psychological therapy with patients. The results revealed high levels of consensus in areas relating to communication style, contracting, quality and value, environment, emotional differences, effort, and technological aspects of engaging clients in this way. Participants reported similar views for therapies delivered via telephone and videoconferencing.

The finding that ellipsis (utterances indicating listening) should be used to supplement communication when working remotely suggests that therapists may be seeking to compensate for the lack of visual listening cues that would be present in face‐to‐face therapy (Sayers, 2021). Although the patient may be able to see the therapist's facial expressions in video therapy, visual information associated with body posture and hand gestures may not be as accessible. The fact that therapists saw the importance of explaining the function of silence in telephone therapy also relates to the lack of visual cues and suggests that therapists can use good verbal communication to compensate for missing information. In the absence of visual cues, therapists may feel the need to explicitly name that being silent does not mean that they are not engaged.

The findings that participants agreed about the need to seek regular patient feedback in telephone therapy and discuss the value of remote sessions in the case of both therapies delivered by telephone or videoconferencing therapies may reflect therapists' own concerns about the value of remote interventions identified in previous research (Full et al., 2024; James et al., 2022; Kotera et al., 2021). Despite the finding that remote synchronous therapy generally demonstrates similar treatment outcomes and patient satisfaction when compared with face‐to‐face therapy (Brenes et al., 2011; Greenwood et al., 2022; Thomas et al., 2021), the absence of visual cues in telephone therapy and the reduced visual cues in therapy delivered via videoconferencing may impact on the therapist's confidence in the alliance.

Not surprisingly, the importance of providing the patient with a choice of therapy medium was a high consensus statement and reflects the positive correlation between increased choice, therapeutic alliance and outcomes in mental health services (Stanhope et al., 2013). The finding also relates to the consensus statement regarding the importance of discussing the limitations of remote therapies with patients. Awareness raising about the potential limitations of remote therapies enables patients to make informed choice about whether or not to engage in remote interventions. These discussions should, however, include research evidence suggesting comparable outcomes in remote and online therapies (Brenes et al., 2011; Thomas et al., 2021) and not reflect therapists' own unfounded anxieties about remote interventions.

The finding that participants agreed that it was important to name and discuss difficulties in alliance as they develop is reflective of good practice across all modes of therapy delivery and relates to the process of rupture and repair within the therapeutic alliance typically documented within face‐to‐face therapy (Marquardt, 2021; Safran et al., 2011). However, in remote interventions, some difficulties may occur more often than they would in face‐to‐face therapy, reflected by the importance therapists placed on the need to develop plans in the event of difficulties in therapy arising. For example, in Round 1 participants raised concerns about the impact of unintentionally interrupting patients and reached consensus around the importance of naming this when it happens. Previous literature and guidance in relation to remote therapies similarly highlight the benefits of speaking more slowly and explicitly using turn‐taking to help reduce the impact of interruption on the therapeutic relationship (Gros et al., 2013; Wootton et al., 2020).

In the case of therapy delivered via videoconferencing, therapists reached consensus about the need for a conversation with patients about impact of seeing one's own face on screen. This phenomenon is unique to videoconferencing and researchers are investigating how it impacts on the experience of therapy, which may be particularly important amongst certain patient groups, such as those with low self‐esteem or body dysmorphia (Grignoli et al., 2021; Pfund et al., 2020; Pikoos et al., 2021). Recommendations for dealing with the potentially negative effect of this phenomenon include using the software to turn off ‘self‐view’, using gallery view during group work (Hart et al., 2022) or even switching to telephone therapy (Grignoli et al., 2021). In the telephone medium, consensus was gained concerning the importance of discussing the sense of anonymity that can arise in the context of telephone therapy. In Round 1, participants raised concerns about anonymity due to lack of visual cues and how this may impact negatively on the patient and therapist getting to know each other. In contrast, there is evidence of the benefits of anonymity in therapy, such as a lessened sense of threat or shame which may lead to increased feelings of safety and comfort, and more rapid and intimate disclosure and consequently, stronger therapeutic bonds (Dhesi et al., 2022; Lingley‐Pottie & McGrath, 2007).

Consensus statements across both media of therapy delivery highlighted the importance of ensuring that the space in which patients access therapy is appropriate, confidential, and safe, and highlighted the importance of noticing and naming distractions within the patient's environment. These environmental conditions are considered equally important for in‐person therapy (Association of Child Psychotherapists, 2021), but when working remotely, they are likely to be more difficult to control as the patient is not in a space that the clinician has a high degree of control over. In Round 1, clinicians reported some patients accessing therapy from home or whilst driving or shopping, all of which are spaces in which some, or all these conditions, cannot be met. However, despite reported concerns about inappropriate therapy environments when delivering remote therapies, clinicians have identified benefits of engaging with a patient in their own environment. For example, if using videoconferencing, seeing the patient's environment may add helpful contextual information for assessments and formulation (Lindsay et al., 2017). If the patient is within their own environment, this may also alter the power balance between patient and therapist, leading to improved patient reported alliance ratings (Simpson et al., 2005). The finding that therapists agreed that it is important to provide a consistent and noise‐free background within therapy delivered by video conferencing may reflect the importance therapists place on creating consistency and safety within in‐person therapy rooms (Sinclair, 2020).

The finding that therapists did not agree about the importance of additional self‐care whilst engaging in remote interventions is surprising as it contrasts with the much talked about notion of ‘Zoom fatigue’ (Sklar, 2020; Wiederhold, 2020) and previous research suggesting that therapists report feeling more tired delivering online therapy compared with therapy within face‐to‐face settings (Aafjes‐van Doorn et al., 2021). It also contrasts with the finding that therapists agreed that telephone therapy can be more effortful possibly resulting from the need to attend more to non‐visual verbal cues (Irvine et al., 2020). High and demanding workloads may mean that despite increased fatigue associated with online working, therapists do not have sufficient time to reflect on the need for or attend to their self‐care needs.

Participants agreed that using software to share screens was important in therapy delivered via video. This finding may reflect therapists' efforts to emulate face‐to‐face therapies where therapists and patients routinely looked at information together or draw out visual representations of a patient's difficulties (Jefferis et al., 2021; Redhead et al., 2015). The finding that therapists also agreed about the need to familiarize patients with videoconferencing technology is consistent with findings in the literature that limited digital literacy can increase barriers to forming successful therapeutic relationships (Benz et al., 2022). Implementing support and training to help patients familiarize themselves with the technology involved (Puspitasari et al., 2021) may negate frustrations that would otherwise interfere with alliance building. However, most of these concerns about technology that therapists identified in Round 1 were out of their immediate control (e.g. speed of a client's internet connection).

In the telephone medium alone, it was considered important to send additional materials between sessions and may have been considered particularly important in the absence of opportunities to share information visually during sessions. Research shows that engagement with, and completion of, homework outside the therapy room improves therapeutic outcomes (Lebeau et al., 2013). The same appears to be true when working via telephone (e.g. Aguilera et al., 2018) and can support clients to monitor progress towards their goals, practice therapy techniques, and provide feedback to clinicians within sessions (Leach & Christensen, 2006).

### Strengths and limitations

Some of the major strengths of Delphi methodology lie in its ability to access the opinions of multiple participants without the constraints of geographical location (Donohoe & Needham, 2009). The anonymity of online Delphi methods also allows for independent thought, unmarred by the potential for dominant voices that may impact other data collection methods such as focus groups (Dalkey & Helmer, 1963). The methodology utilizes both qualitative and quantitative data, allowing for the richness of the experience of participants to be concentrated in a clear and meaningful way (Iqbal & Pipon‐Young, 2009). The therapeutic alliance itself is a complex concept with numerous overlapping and interrelated conceptualizations that vary across therapeutic approaches and between clinicians (Hougaard, 1994). We were able to integrate and synthesize the views of clinicians with varying backgrounds and levels of experience, meaning that findings can be applied to therapeutic work across multiple settings and to people at different stages of their careers. However, whilst these findings represent a broad understanding of steps that can be taken to establish and maintain the therapeutic alliance during remote synchronous therapy, the findings may be over‐generalized and should therefore be applied with caution to more specific patient groups. Furthermore, other than occupation, we did not collect sociodemographic information about our participants. The omission limits the ability to assess the representativeness of our sample and therefore the generalizability of our findings.

Participant attrition in Delphi can vary between 20% and 87% in Delphi studies (Hall et al., 2018). In this study, the attrition rate reached 52.35% in Round 3. Therefore, the final round results do not reflect the views of all participants who originally contributed their responses in Round 1 from which the statements were generated. A further limitation to the Delphi methodology is the inherent balance that is required between attempting to ensure all statements meet consensus, be that agreement or disagreement, whilst completing the process in a timely manner that maintains participant engagement (Hasson et al., 2000). Consensus was reached for less than half of the presented statements (49.21%). A contributing factor to this may be that the consensus level was set at 80%, which is comparatively high compared with other research employing the Delphi methodology in which consensus threshold ranges from 50% to 97%, with a mean of 75% (Diamond et al., 2014). We felt that the higher threshold was necessary in the event the findings are used to implement recommendations in clinical practice. It may be that if further iterative rounds were carried out, more statements may have reached consensus. Nevertheless, the fact that several statements did not reach consensus is an inherent limitation.

### Clinical implications and future research

Whilst guidelines are available for conducting therapy remotely (BPS, 2020), empirical support for the process of establishing and maintaining the therapeutic alliance remotely is limited (James et al., 2022). The findings of this study represent a broad group of clinicians with varying levels of experience, meaning that the data provide a valuable source of information in the development of guidelines for establishing and maintaining therapeutic relationships within remote synchronous therapy. Table 2 summarizes preliminary practice recommendations derived from statements that have been deemed to be important across both videoconferencing and telephone media, followed by those applying to videoconferencing only and finally telephone only.

**TABLE 2:** Practice recommendations differentiated by remote medium.

Videoconferencing and Telephone‐delivery	Prioritize active listening sounds to ensure empathy is clearly communicated Offer a choice of therapy medium where possible as this will likely positively impact the therapeutic alliance Developing a plan during the contracting phase to manage situations where technology fails. This may be particularly important in the early stages of therapy when the patient is new to the technology and particularly relevant for clinicians who have less experience/training in therapy via remote means Explicitly discuss the importance of having an appropriate, confidential, and safe environment with the patient. It is important the patient feels safe in their environment and that they can engage in therapy without significant distractions. Be clear about what environments are considered inappropriate for therapy (e.g. café, supermarket)
Videoconferencing	Discuss the impact of the patient seeing their own face on screen and make any adjustments necessary, such as encouraging the patient to turn off self‐view, practicing mindfulness to draw attention to where patients are focusing their attention, or promoting self‐compassion. Maintain consistency of the clinician's own environment (e.g. background) to promote a sense of safety and reduce distractions for the patient Consider using aspects of videoconferencing software that allow for visual sharing of information, such as using a shared ‘whiteboard’ or sharing their screen. This may be particularly important when developing collaborative case formulations Clinicians should seek out training or sufficiently familiarize themselves with videoconferencing software prior to using this medium to engage patients in therapy Provide resources to support patients to develop an adequate level of understanding around how to engage with videoconferencing technology prior to commencing therapy. This may help to negate unnecessary frustration in the early stages of therapy
Telephone	Discuss the use of silence and how this might be managed or what this might mean Hold in mind the impact that anonymity may help and/or hinder the therapeutic alliance during telephone therapy. Whilst relationship building may be more rapid, there may also be information lost due to lack of visual information Consider patient motivations to opt for telephone therapy if they had the opportunity to choose a medium in which their identity would be more evident Be mindful that telephone therapy may require increased effort levels, which could lead to fatigue. Practice additional self‐care to avoid compassion fatigue and ensure that the quality of therapy and the therapeutic relationship is maintained Provide additional materials to support the patient between sessions, ideally materials that provide a visual representation of therapeutic concepts or processes that is otherwise unavailable via telephone

The preliminary practice recommendations are based on clinician consensus, which is an important source of knowledge in its own right, but it also provides researchers seeking to explore predictors of alliance in remote therapy with information about which variables to prioritize investigating. Further research should seek to understand the effect that training, experience, and familiarization with remote mediums of working have on clinicians' perceptions of establishing and maintaining therapeutic alliances over videoconferencing and telephone. As this study did not ask clinicians about the clinical populations that they work with, it would also be useful to further explore whether clinicians' experience of remote therapeutic alliances differs when working with specific populations. The patient and therapist perceptions of alliance are not always highly correlated (Tryon et al., 2008), meaning that the two parties may value different aspects of the therapeutic relationship. As such, it is important to also explore patients' perceptions of the alliance within remote therapies. Using a Delphi methodology to explore this topic would provide an opportunity to directly compare the patients' perspective with the therapists' perspectives reported in this article.

## CONCLUSIONS

In summary, this study provides a summary of factors that clinicians agree are important for developing a therapeutic alliance within remote therapies delivered either by telephone or videoconferencing. Future research is needed to establish whether these factors, which are based on clinical experience, are supported by empirical evidence. However, clinicians who have had to navigate the rapid rise in online delivery of therapy have valuable insights which warrant sharing amongst communities of practicing therapists and those in training. Identifying factors which a significant number of therapists agree are important also helps guide researchers in identifying factors that warrant further investigation through empirical studies.

## AUTHOR CONTRIBUTIONS

**Sam Tyrrell:** Conceptualization; methodology; writing – original draft; formal analysis; project administration; data curation. **Sandra Bucci:** Conceptualization; writing – review and editing; supervision. **Katherine Berry:** Conceptualization; writing – review and editing; supervision.

## FUNDING INFORMATION

National Institute of Health Care Research. SB was supported by a research professorship NIHR300794 from the National Institute for Health and Care Research and KB and SB were supported by the Manchester Biomedical Research Centre (NIHR203308). The views expressed are those of the authors and note necessarily those of the NIHR or the Department of health and Social Care.

## CONFLICT OF INTEREST STATEMENT

None to declare.

## Supporting information


Appendix S1

## Data Availability

Data are available from the lead author upon reasonable request.
