# Supplementary material for: The therapeutic alliance during remotely delivered therapy: A Delphi study with health professionals
Source: Br J Clin Psychol. 2025 Apr 4;64(4):857–72. doi: 10.1111/bjc.12544 (PMC12506956; doi:10.1111/bjc.12544)
Supplement: Supplementary file 1 — Appendix S1 [file BJC-64-857-s001.docx]

Supplementary material: Statements generated from Round 1.

| **Category** | **Statement** |  |
| --- | --- | --- |
| Videoconferencing | |  |
| Communication Style | 1. It is important to ensure that your hands are visible on-screen during therapy when working via online videoconferencing. |  |
|  | 1. It is important to use more listening sounds like ‘hmm’ and ‘ahh’ when working via online videoconferencing compared to face to face therapy. |  |
|  | 1. It is important to use more self-disclosure to develop and maintain the therapeutic alliance when working via online videoconferencing compared to face-to-face therapy. |  |
|  | 1. It is important to use more humour to develop and maintain the therapeutic alliance when working via online videoconferencing compared to face-to-face therapy. |  |
|  | 1. It is important to slow down the pace of therapy when working via online videoconferencing compared to face-to-face therapy. |  |
|  | 1. It is important to discuss the function of therapeutic silence explicitly during therapy when working via online videoconferencing. |  |
|  | 1. It is important to discuss how to manage silence or pauses when working via online videoconferencing. |  |
|  | 1. It is important to more actively ensure that the client feels ‘held in mind’ during therapy via online videoconferencing compared to face-to-face therapy, such as referring to an interest or event they mentioned in a previous session. |  |
|  | 1. It is important to seek more regular feedback about the client’s experience of therapy when working via online videoconferencing compared to face-to-face therapy. |  |
| Contracting | 1. It is important to allow the client to make the choice to access therapy via online videoconferencing as an alternative to face-to-face therapy. |  |
|  | 1. It is more important to establish clearer goals when working via online videoconferencing compared to face-to-face therapy. |  |
|  | 1. It is important to take time to discuss the limitations of therapy via online videoconferencing with the client at the start of online therapy. |  |
|  | 1. It is important to continue to name difficulties with therapy via online videoconferencing as they occur. |  |
|  | 1. It is important to explicitly acknowledge unintentionally interrupting clients during therapy via online videoconferencing. |  |
|  | 1. It is important to develop a plan for when technology goes wrong during therapy when working via online videoconferencing. |  |
|  | 1. It is even more important to establish a consistent slot for therapy (e.g. same day and time weekly) when using online videoconferencing, compared to face-to-face therapy. |  |
|  | 1. It is beneficial to the therapeutic alliance to meet face to face for the first therapy session before switching to therapy via online videoconferencing. |  |
| Emotional differences | 1. It is particularly important to discuss ‘readiness’ for therapy with a client when working via online videoconferencing. |  |
|  | 1. It is important to acknowledge with a client that therapy via online videoconferencing can sometimes cause additional anxiety compared to face-to-face therapy. |  |
|  | 1. It is important to discuss the potential impact of seeing one’s own face on screen during therapy via online videoconferencing. |  |
| Quality/Value | 1. It is important to discuss with the client whether they feel that therapy via online videoconferencing is as valuable as face-to-face therapy. |  |
| Environment | 1. It is important to discuss the appropriateness of the environment in which the client accesses therapy via online videoconferencing. |  |
|  | 1. It is more important to discuss the confidentiality of the therapy environment when working via online videoconferencing compared to face to face. |  |
|  | 1. It is important to comment on your client’s environment, or things you notice in their environment, during therapy via online videoconferencing. |  |
|  | 1. It is important to ensure that your (the professional) environment is consistent (i.e. in the same location with the same background) when engaging in therapy via online videoconferencing. |  |
|  | 1. It is important to discuss whether the client feels safe in the environment in which they are accessing therapy via online videoconferencing. |  |
|  | 1. It is important to make time to for ‘non-therapy talk’ during therapy sessions when working via online videoconferencing as the conversations between waiting room and therapy room are lost during remote work. |  |
|  | 1. It is more important to notice and name distractions in the client’s environment when working via online videoconferencing compared to face-to-face therapy. |  |
| Effort | 1. It is important to acknowledge with the client that therapy via online videoconferencing is often more effortful than when working face to face. |  |
|  | 1. It is important to ensure that additional time is made for therapist self-care when working via online videoconferencing, compared to working face to face. |  |
| Technology | 1. It is important to ensure that screen share is used to supplement communication during therapy via online videoconferencing, such as when drawing out formulations or sharing resources. |  |
|  | 1. It is important to arrange additional time prior to the start of therapy via online videoconferencing to ensure that clients have the knowledge to use the technology to access therapy via   videoconferencing. |  |
|  | 1. It is more important to send additional materials for use between therapy sessions via email (or an appropriate alternative) when working via online videoconferencing compared to providing   materials for use between sessions during face-to-face therapy. |  |
| **Telephone** | |  |
| Communication style | 1. It is important to use more listening sounds like ‘hmm’ and ‘ahh’ when working via telephone. |  |
|  | 1. It is important to use more self-disclosure to develop and maintain the therapeutic alliance when working via telephone compared to face-to-face therapy. |  |
|  | 1. It is important to use more humour to develop and maintain the therapeutic alliance when working via telephone compared to face-to-face therapy. |  |
|  | 1. It is important to slow down the pace of therapy when working via telephone compared to face-to-face therapy. |  |
|  | 1. It is important to discuss the function of therapeutic silence explicitly during therapy when working via telephone. |  |
|  | 1. It is important to discuss how to manage silence or pauses when working via online telephone. |  |
|  | 1. It is important to more actively ensure that the client feels ‘held in mind’ during therapy via telephone compared to face-to-face therapy, such as referring to an interest or event they mentioned in a previous session. |  |
|  | 1. It is important to seek more regular feedback about the client’s experience of therapy when working via telephone compared to face-to-face therapy. |  |
| Contracting | 1. It is important to allow the client to make the choice to access therapy via telephone as an alternative to face-to-face therapy. |  |
|  | 1. It is more important to establish clearer goals when working via telephone compared to face-to-face therapy. |  |
|  | 1. It is important to take time to discuss the limitations of therapy via telephone with the client at the start of online therapy. |  |
|  | 1. It is important to continue to name difficulties with therapy via telephone as they occur. |  |
|  | 1. It is important to explicitly acknowledge unintentionally interrupting clients during therapy via telephone. |  |
|  | 1. It is important to develop a plan for when technology goes wrong during therapy when working via telephone. |  |
|  | 1. It is even more important to establish a consistent slot for therapy (e.g. same day and time weekly) when working via telephone, compared to face-to-face therapy. |  |
|  | 1. It is beneficial to the therapeutic alliance to meet face to face for the first therapy session before switching to therapy via telephone |  |
| Emotional differences | 1. It is particularly important to discuss ‘readiness’ for therapy with a client when working via telephone. |  |
|  | 1. It is important to acknowledge with a client that therapy via telephone can sometimes cause additional anxiety compared to face-to-face therapy. |  |
|  | 1. It is important to discuss with the client the anonymity that comes when working via telephone which is often not present when working face to face or via videoconferencing software. |  |
| Quality/Value | 1. It is important to discuss with the client whether they feel that therapy via telephone is as valuable as face-to-face therapy. |  |
| Environment | 1. It is important to discuss the appropriateness of the environment in which the client accesses therapy via telephone. |  |
|  | 1. It is more important to discuss the confidentiality of the therapy environment when working via telephone compared to face to face. |  |
|  | 1. It is important to comment on your client’s environment, or things you hear in their environment, during therapy via telephone. |  |
|  | 1. It is important to ensure that your (the professional) environment is consistent (i.e. in the same place with the same level of noise etc) when engaging in therapy via telephone. |  |
|  | 1. It is important to discuss whether the client feels safe in the environment in which they are accessing therapy via telephone. |  |
|  | 1. It is important to make time to for ‘non-therapy talk’ during therapy sessions when working via telephone as the conversations between waiting room and therapy room are lost during remote work. |  |
|  | 1. It is more important to notice and name distractions in the client’s environment when working via telephone compared to face-to-face therapy. |  |
| Effort | 1. It is important to acknowledge with the client that therapy via telephone is often more effortful than when working face to face. |  |
|  | 1. It is important to ensure that additional time is made for therapist self-care when working via telephone, compared to working face to face. |  |
| Technology | 1. It is more important to send additional materials for use between therapy sessions via email (or an appropriate alternative) when working via telephone compared to providing materials for use   between sessions during face-to-face therapy. |  |
